# Diving In: Experiential Learning about Research

**DOI:** 10.5811/westjem.2019.7.44526

**Published:** 2019-12-10

**Authors:** Benjamin H. Schnapp

**Affiliations:** University of Wisconsin School of Medicine and Public Health, BerbeeWalsh Department of Emergency Medicine, Madison, Wisconsin

“I hate research,” I hear many junior physicians say. They have likely received the advice given to many novices looking to get started with scholarly work: find an experienced researcher and jump on to one of their hulking projects for a brief moment of chart abstraction, recruitment of subjects, or data analysis. Certainly, finding mentors with a track record of success is laudable, and this approach does “check the box”—fulfilling the scholarly project requirement to graduate from residency or filling up a curriculum vitae for academic promotion. It is perhaps not surprising, however, that staring at endless spreadsheets of data, stripped of nearly all context, can dissuade potential budding scholars. This approach is akin to dipping only a toe in the waters of scholarly inquiry, allowing potential researchers to experience only one tiny piece of a much larger whole and missing the chance to foster a spirit of engagement with the scientific process.

Research sprang to life for me when a mentor offered me the opportunity to craft a research project by his side. No longer was I a passive participant, executing a master plan that I didn’t really understand. Instead, I got to ask my own questions, realize that many of them had already been answered, and then keep asking questions and refining. I got to devise my own plan to test my question and then go back to the drawing board when those plans didn’t work. I got to experience the terror of staring desperately at a blank screen, trying to start crafting a manuscript. While none of these experiences sound appealing on first look, to me it was the difference between being the backseat passenger in the car and sitting down behind the wheel: There’s no comparison to that feeling of being in charge of your own research destiny.

Kolb’s experiential learning cycle suggests why it is these immersive experiences that offer the most educational yield. The cycle describes four phases of learning, “concrete experience,” “reflective observation,” “abstract conceptualization,” and “active experimentation.” Learning occurs when all four phases have been completed.^1^ Tackling someone else’s project certainly offers the “concrete experience” of learning how to recruit a subject or code data, essential components of successfully accomplishing a research project. It may even foster opportunities for “reflective observation” on what was learned. However, only the opportunity to pursue your own scholarly interests independently offers the opportunity for the generation of novel ideas through “abstract conceptualization” and the refinement of those ideas in the real world through “active experimentation,” and therefore the ability to complete the learning cycle and cement learning about scholarly inquiry.

When thinking about Kolb’s “active experimentation,” it can be easy to overlook the “active” component of this stage in the learning cycle. Junior researchers can sometimes be seduced by their one big research project idea or the outstanding multicenter studies they read in the literature, becoming frustrated and spinning their wheels unproductively when they begin to hit unanticipated roadblocks. Scholarly work is much like riding a bike, however; you cannot remove your training wheels and expect to compete in the Tour de France. My first research projects involved only the residents at my own institution and took place over a single year. The projects were never bound for *The New England Journal of Medicine*, but they established a solid foundation that I could build on with subsequent projects. There is real value in “thinking small” when starting out with research: picking a project with a shorter timeline or a more limited scope allows for more rapid experience with each stage of Kolb’s learning cycle and more opportunities for experimentation and growing confidence.

Active experimentation also cannot be accomplished in a vacuum. Attempting to dive into the research pool without any swimming lessons or lifeguards deprives you of chances to benefit from the vast array of wisdom available from experienced researchers. A mentor can offer concrete help - an additional article from the literature for framing your study or modifications for your data analysis. They can also help navigate more abstract issues, such as clarifying your research question, helping you stay motivated to complete your manuscript, as well as guiding you on next steps when you’ve completed your project.

Ongoing mentorship while learning to become a researcher is supported by Bloom’s mastery learning theory. The theory states that with the proper supervision and enough repetitions, nearly anyone can achieve a high standard of performance.^2^ With an instructor to help guide the work and ensure that new ideas are on target, an apprenticing researcher can achieve true deliberate practice, rather than continuing to make the same mistakes again and again. Mentors allows junior researchers to train with good habits as they move through the steps of completing their projects.^3^ Even with mentorship however, it can be daunting to embark on new projects on your own. When struggling through a particularly vexatious problem or just trying to stay motivated to keep plugging away, research can seem isolating, as though you are the only person struggling with how to calculate a confidence interval or write a compelling introduction.

Seeking out a peer group of other junior researchers can offer insight, support and opportunities for collaboration. If an obvious peer group doesn’t exist within your own institution, the Internet offers the opportunity to instantly partner with colleagues at distant sites and benefit from networking from afar via Twitter or Slack. Further, creating collaborations outside your institution offers the chance to eventually expand your research to multiple sites, increasing its potential impact. Wenger would call these peer groups focused on learning and support “communities of practice.” Members of these groups engage in collective education in a shared domain,^4^ in this case, research. Importantly, these communities allow for the participation of new members via “legitimate peripheral participation”; so it is not necessary to be a seasoned expert or full participant right away – you can work your way into the community of learners at a pace that feels comfortable to you, benefitting along the way from the wisdom of the crowd.

I’ve picked up countless lessons along the way from the projects I’ve completed so far. The importance of identifying allies within your institution, teaming up with accountable collaborators, choosing accomplishable goals, getting a statistician on board early, and writing your manuscript as you go are but a few of the tips that I take with me, and there is undoubtedly so much more to learn as I step into my next projects. But don’t just take advice from my experiences. Dive in and turn your idea into the next amazing research project and pick up your own lessons along the way. You’ll be happily swimming the scholarly seas in no time.
